# Does laparoscopic treatment of deep endometriosis improve sexual dysfunction 

**DOI:** 10.22088/cjim.14.2.267

**Published:** 2023

**Authors:** Abolfazl Mehdizadehkashi, Shahla Chaichian, Samaneh Rokhgireh, Kobra Tahermanesh, Shahla Mirgaloybayat, Reza Saadat Mostafavi, Sepideh Khodaverdi, Marziyeh Ajdary, Mahin Ahmadi Pishkuhi

**Affiliations:** 1 Endometriosis Research Center, Iran University of Medical Sciences, Tehran, Iran; 2 Department of Radiology, Rasool Hospital, Tehran, Iran; 3 Pars Advanced and Minimally Invasive Medical Manners Research Center, Pars Hospital, Tehran, Iran

**Keywords:** Endometriosis, Laparoscopy, Sexual dysfunction

## Abstract

**Background:**

Endometriosis is one of the common gynecological problems during the reproductive years, affecting the quality of life, fertility, and sexual function of women. It is known that sexual dysfunction and quality of life are interrelated. Therefore, this study aimed to evaluate the effect of resection of endometriosis lesions via laparoscopic surgery on the improvement of sexual dysfunction in women with endometriosis.

**Methods:**

This clinical trial was performed on 30 patients with endometriosis. The Female Sexual Function Index, Endometriosis Health Profile-30, and Visual Analog Scale were completed for the patients before laparoscopic surgery and three, six, and 12 months after surgery. The results were examined and compared before and after the intervention using the ANOVA test.

**Results:**

The present results showed that the mean pain score of the patients (dysmenorrhea, dyspareunia, and pelvic pain) was significant after laparoscopic surgery (P<0.005). The female sexual function improved after laparoscopic surgery compared to the preoperative phase, and changes in the domains of psychological stimulation, humidity, and sexual orgasm were significant (P<0.005). Moreover, the female quality of life scores increased in all dimensions compared to the preoperative phase, although these improvements were not statistically significant.

**Conclusion:**

The present results revealed that laparoscopic surgery is an effective treatment, leading to a considerable improvement in female sexual function.

Endometriosis is a disease characterized by the presence of endometrial glands and stroma outside the uterine cavity (1). This disease is one of the common gynecological problems during the reproductive years (2), affecting women’s quality of life and fertility (3). About 10% of women of childbearing age suffer from pelvic pain and infertility due to endometriosis (4). 

Endometriosis affects women of all social and racial classes throughout the reproductive period (from the onset of menstruation until menopause). Laparoscopy is one of the available treatments for endometriosis, in which a telescope-like instrument, called a laparoscope, is inserted through an abdominal incision to observe the reproductive organs. Laparoscopy can be performed to identify various conditions, such as tubal damage, endometriosis, pelvic adhesion, and tuberculosis, which can cause infertility. The growing tendency toward the use of minimally invasive gynecological surgeries has increased the number of patients undergoing laparoscopy. Besides incisional pain, laparoscopic pain has been attributed to peritoneal inflammation, pneumoperitoneum, and pelvic or abdominal rupture. Peritoneal manipulation leads to visceral stimulation and transmission of messages through the enteric nervous network. Pain may be slightly diffuse, localized, or recurrent. 

Visceral pain following laparoscopic surgery can be as severe as incisional pain due to gas murmurs and manipulation of abdominal structures. Moreover, visceral stimulation can be associated with autonomic reflexes, such as nausea and vomiting. Endometriosis during the course of infection can have various symptoms (5). Dyspareunia is one of the most common symptoms, which severely affects women’s sex life quality. Endometrial implant infiltration of more than 5 mm is known as deep infiltrating endometriosis (DIE), involving the fibromuscular tissue, endometrial glands, and stroma. DIE is strongly associated with the symptoms of severe pelvic pain (6, 7). 

Evaluation of sexual status in patients with DIE indicated their poor sexual function, including sexual dissatisfaction and anorgasmia. The analysis of the effects of endometriosis lesions and symptoms on sexual function revealed that deep and vaginal endometriosis clearly affected women’s sexual activity (8). The present study aimed to determine the effect of laparoscopic treatment for deep endometriosis on sexual dysfunction, quality of life, and pain in women with endometriosis, referred to Rasoul Akram Hospital in Tehran, Iran, in 2016.

## Methods

In this clinical trial, according to the inclusion criteria, 30 endometriosis patients with informed consent were included during 2016-2018. Before the intervention, the Female Sexual Function Index (FSFI), Endometriosis Health Profile-30 (EHP-30), and Visual Analog Scale (VAS) were completed by the participants. They underwent laparoscopic surgery after completing the questionnaires.

 The inclusion criteria in this study were as follows: being married; being sexually active; experience of sexual dysfunction in the past six months; confirmed diagnosis of DE at the time of laparoscopy early diagnosis of endometriosis; chronic pelvic pain associated with endometriosis; completing an informed consent form before the study; lack of pelvic pain originating from other organs (e.g., gastrointestinal, urinary, and genital causes); no use of gonadotropins or other hormonal drugs in the last three months; no gynecological malignancies; no uterine fibroids on the preoperative ultrasound; no underlying diseases (e.g., cardiovascular, respiratory, renal, hematological, hepatic, neurological, or psychological diseases); no pregnancy; patients with no evidence of endometriosis or had stage I, II and III endometriosis diseases were excluded from this study; and no willingness to continue the study. Three, six, and twelve months after laparoscopic surgery, the patients were examined again, and their sexual function, quality of life, and average pain were assessed. The results were compared before and after the intervention.

**Sampling method and sample size: **All patients with a diagnosis of endometriosis and sexual dysfunction, referred to the gynecology ward of Rasoul Akram Hospital for laparoscopy during 2016-2019, were included. The sample size was estimated at 35 (z_1-α/2_=1.96, s=12.6, d=4.24), based on previous studies (9). 

**Data collection methods and tools: **Before and after surgery for pelvic pain, a gynecologist performed a vaginal examination for the evaluation of the extent of disease and pelvic pain score. The data collection tools included two forms of demographic information and gynecological history, along with the FSFI, EHP-30, and VAS questionnaires, the validity, and reliability of which have been reviewed and confirmed in the Iranian population. In a study by Rosen et al. in 2000, the reliability coefficient of FSFI was estimated at 0.89 (10), while in a study by Mohammadi et al. in 2008, it was measured to be 0.7 (11). In Iran, FSFI was validated by Mohammadi et al. in 2008. The validity and reliability coefficients of this questionnaire were 0.66 and 0.70, respectively (11). Moreover, EHP-30, with validity and reliability coefficients of 0.93 and 0.90, respectively, was also validated by Nojomi et al. in Iran in 2011 (12). Finally, the VAS, with reliability and validity coefficients of 0.73 and 0.79, respectively, was validated by Rezaei et al. in Iran in 2011 (13).

**Ethical considerations: **This study was carried out with the approval of the Research Council of the Medical School and the Ethics Committee of Iran University of Medical Sciences, Tehran, Iran. It was also registered in the Iranian Registry of Clinical Trials (IRCT) (code: IRCT20150817023666N8). The participants were allowed to withdraw from the study at any time by signing a written informed consent form. The patients were assured that their personal information would remain confidential.

**Data analysis: **SPSS version 22 was used to analyze the data. Frequency (percentage) was measured to summarize qualitative variables, and the ANOVA test was used to compare the results before and after the intervention. In all analyses, a P-value less than 0.05 was considered statistically significant.

## Results

In this study, a total of 35 patients with endometriosis were included according to the sample size. However, four patients were excluded (one due to pregnancy and three due to unwillingness to cooperate). Finally, the study was carried out among 31 participants. According to the results, the mean age of endometriosis women was 35.06±6.3 years. The participants' demographic information is presented in table 1. 

**Table 1:** The demographic characteristics of patients with endometriosis before laparoscopic surgery

**Variables**	**Number**	**Minimum**	**Maximum**	**Mean**	**SD**
**Age (year)**	31	26.00	50.00	35.0645	6.37148
**Gravidity (Number)**	31	.00	5.00	1.1613	1.34404
**Parity ** **(Number)**	31	.00	3.00	.8710	.88476
**Weight (kg)**	31	49.00	87.00	67.2581	10.01322
**Height (cm)**	31	150.00	180.00	162.0323	7.04502
**BMI (** **kg/m** ^2^ **)**	31	18.00	33.98	25.6344	3.59025
**Education, Number (%)**	Middle school	7	22.6	-	-
	High school diploma	14	45.2	-	-
	Higher education	10	32.3	-	-
**Occupational status, Number (%)**	Housewife	25	80.6	-	-
	Employee	5	16.1	-	-
	Self-employed	1	3.2	-	-
**Marital status, Number (%)**	Married	29	93.5	-	-
	Divorced	2	6.5	-	-
**Contraceptive method, Number (%)**	Contraceptive pills	2	6.5	-	-
	Condoms	2	6.5	-	-
	IUD	2	6.5	-	-
	Withdrawal	12	38.7	-	-
	None	13	41.9	-	-
**Ethnicity, Number (%)**	Turk	12	38.7	-	-
	Lur	2	6.5	-	-
	Gilaki	3	9.7	-	-
	Fars	6	19.4	-	-
	Baluch	2	6.5	-	-
	Kurd	2	6.5	-	-
	Arab	2	6.5	-	-
	Mazani	1	3.2	-	-
	Others	1	3.2	-	-
**Medications for endometriosis, Number (%)**	Yes	13	41.9	-	-
	No	18	58.1	-	-
**History of surgery for endometriosis, Number (%)**	Yes	19	61.3	-	-
	No	12	38.7	-	-

BMI: Body mass index

The results of the ANOVA test showed that the mean pain scores of the patients (i.e., dysmenorrhea, dyspareunia, dysuria, pelvic pain, and dyskinesia) decreased after laparoscopy. The reduction in the scores of dysmenorrhea, dyspareunia, and pelvic pain was significant after laparoscopy (P<0.005) (table 2), whereas the decrease in dysuria and dyskinesia was non-significant. The mean scores of different domains of female sexual function before surgery and during the follow-up are presented in table 3.

 According to the results, women's sexual function scores improved in all domains after laparoscopic surgery; however, only changes in the domains of psychological stimulation, vaginal moisture, and sexual orgasm were significant (P<0.005). 

The ANOVA test results showed that the quality of life scores of women improved in all domains compared to the preoperative phase; however, these improvements were not statistically significant (table 4).

**Table 2:** Comparison of the pain scores of women with endometriosis before laparoscopy and three, six, and 12 months after surgery

**Pain score** **(0-10)**	**Before surgery**	**Three months after surgery**	**Six months after surgery**	**Twelve months after surgery**	P-value*
	**Mean (SD)**				
**Dysmenorrhea**	7.85±2.57	2.66±1.11	3.51±2.01	3.12±1.74	<0.001
**Dyspareunia**	4.58±3.25	2.58±1.83	2.70±1.79	2.74±1.87	0.0020
**Dysuria**	2.12±2.96	1.90±2.53	1.58±1.66	1.61±2.13	0.7750
**Pelvic pain**	5.03±4.04	3.00±2.22	2.32±1.83	2.19±1.66	<0.001
**Dyschezia**	2.64±3.07	1.70±2.01	1.48±1.74	1.41±1.58	0.105

**Table 3:** Comparison of changes in the sexual function scores of women with endometriosis before laparoscopy and three, six, and 12 months after surgery

**FSFI domains**	**Before surgery** **Mean±SD**	**Three months after surgery** **Mean±SD**	**Six months after surgery** **Mean±SD**	**Twelve months after surgery** **Mean±SD**	P-value*
**Libido**	2.24±0.65	2.25±0.68	2.45±0.85	2.45±0.85	0.542
**Psychological stimulation**	1.89±1.11	1.96±1.12	2.70±0.77	2.70±0.77	<0.001
**Vaginal moisture**	1.58±1.00	1.70±1.04	2.26±0.72	2.28±0.71	0.002
**Sexual orgasm**	2.12±1.44	2.15±1.34	2.76±0.80	2.86±0.75	0.014
**Sexual satisfaction**	2.62±1.41	2.50±1.40	2.95±1.35	3.03±1.29	0.363
**Pain**	2.05±1.56	1.97±1.34	2.37±1.07	2.52±1.03	0.279

**Table 4:** Comparison of changes in the quality of life of endometriosis patients before laparoscopy and 3, 6, and 12 months after surgery

Domains of quality of life in EHP-30 (main section)	Before surgery Mean±SD	Three months after surgery Mean±SD	Six months after surgery Mean±SD	Twelve months after surgery Mean±SD	P-value*
**Pain (questions 1-11)**	2.73±1.04	2.83±1.00	3.07±0.81	3.09±.84	0.339
**Control and disability (questions 12-17)**	2.59±1.52	2.67±1.47	2.98±1.19	3.06±1.22	0.447
**Emotional health (questions 18-23)**	2.82±1.16	2.87±1.12	2.97±.87	3.05±0.81	0.817
**Social support (questions 24-27)**	2.92±1.19	3.06±1.16	3.18±1.11	3.17±1.02	0.784
**Mental self-image (questions 28-30)**	3.40±1.19	3.40±1.12	3.41±1.12	3.52±1.00	0.969
**General health (GHQ) (questions 1-8)**	3.71±0.96	3.20±0.91	3.33±0.68	3.40±0.72	0.107

## Discussion

Human sexuality is a very complex phenomenon, influenced by social, psychological, biological, and hormonal factors. According to previous studies, sex is a vital aspect of quality of life, which is negatively affected by medical conditions and interventions, especially gynecological problems, genital and breast cancers, and infertility (7, 14). Pelvic pain and dyspareunia due to endometriosis reduce the quality of women’s marital relationship, sexual function (8, 14), and mental health (8). Therefore, this study aimed to evaluate the effect of resection of endometriosis lesions via laparoscopic surgery on the rate of sexual dysfunction in women with endometriosis. 

The present results showed that the mean age of women with endometriosis was 35.06±6.3 years. According to the results of the ANOVA test, the mean pain scores of the patients (dysmenorrhea, dyspareunia, dysuria, pelvic pain, and dyskinesia) decreased after laparoscopic surgery; also, the reduction of dysmenorrhea, dyspareunia, and pelvic pain was significant after laparoscopic surgery (P<0.005); however, reductions in dysuria and dyskinesia were not significant. Comparison of the mean scores of different domains of female sexual function before laparoscopy and during the follow-up showed that female sexual function improved after laparoscopic surgery compared to the baseline, and changes in the domains of psychological stimulation, vaginal moisture, and orgasm were significant (P<0.005).

One of the most important aspects of marital life is sexual relationships. According to the present study and previous research, this dimension is disrupted in patients with endometriosis due to severe pain during or after intercourse and sexual dysfunction (15). These women may be unable to fulfill their expected role as sexual partners, which can negatively affect the couple's relationship (16). They may experience intercourse pain due to the involvement of uterosacral ligaments, Pouch of Douglas, posterior vaginal fornix, and anterior wall of the rectum (17), affecting their self-esteem and sexual intercourse (18).

The potential sexual consequences of endometriosis warrant further attention. It may be helpful to refer patients to reproductive health professionals or psychologists who provide sexual counseling (5, 19). Generally, living with endometriosis and the side effects of treatments can affect the patient’s mood and lead to feelings of depression and irritability (20). Feelings of disability, lack of social support, employment problems, and communication are among other common problems (21). According to some studies, women also feel guilty about their inability to fulfill their perceived gender roles (e.g., reduced sexual contact and inability to complete household chores). Some women do not feel feminine enough and do not consider themselves as good partners; also, many of them are concerned that their spouses may leave them if they fail to conceive (22). Therefore, employment, social life, and family relationships are all negatively affected by endometriosis (21).

In this regard, a study by Fritz et al. in 2016 showed significant reductions in women’s pain scores during and after sexual intercourse, feelings of guilt toward the sexual partner, and fear of sexual pain in those with peritoneal and deep diffuse endometriosis after laparoscopic surgery (23). Moreover, in 2013, Fritz et al. reported that dyspareunia significantly reduced within 6-12 months after surgery (8). Besides, a study by Dobison et al. in 2013 showed a significant improvement in sexual life after surgery. During the follow-up, significant improvements were observed in the severity of dysmenorrhea, pelvic pain, and intestinal symptoms. In the long-term follow-up of dysmenorrhea, it was found that dyspareunia significantly improved. Recurrent pelvic pain associated with endometriosis was estimated at 13.3%. 

Researchers have concluded that laparoscopic surgery improves sexual life, dyspareunia, and pain in patients with endometriosis in the long run (9). In a study by Mabrouk et al. in 2011, six months after laparoscopic surgery, all women showed significant improvements in all domains of sexual function (P<0.005). However, there was no significant difference in the mean score of sexual function between segmental resection and nodule shaving in patients with deep intestinal endometriosis (P>0.05). There was also no significant difference in the mean score of sexual function between patients who received medical treatment after surgery and the group without treatment (24). 

According to the results of the ANOVA test, the women's quality of life score improved in all domains, compared to the preoperative phase, although this improvement was not significant. In a study by Fritz et al. in 2013, the quality of sexual life did not improve rapidly with dyspareunia in a 12-month follow-up, indicating the persistent effect of dyspareunia on women’s mental health. The main mechanism in dyspareunia is the fear of pain recurrence resulting from previous painful sexual experiences; in other words, the experience of pain and the loss of sexual pleasure are constantly recognized and reinforced in recurrent experiences. This process disrupts the person’s sexual life by creating a cognitive pattern of negative expectations. Besides the lack of cooperation of some patients was one of the serious limitations of the present study, so that in some cases, the researchers had to contact patients and convince them for overcoming the tabue of talking about sexual issues several times.

According to the present results, laparoscopic surgery, as an effective treatment method, could significantly improve women's sexual function. However, for a better description of the impact of laparoscopic surgery on the quality of life and sexual function in endometriosis patients the authors recommend that more studies be designed and maybe better if the researches are focused on the combination of laparoscopic surgery and sexual counseling.
